# Preoccupied and Fearful-Avoidant Attachment Styles May Mediate the Relationship Between Poor Parental Relationship Quality and Sexual Interests in Violence

**DOI:** 10.1007/s10508-025-03183-6

**Published:** 2025-07-18

**Authors:** Ellen Zakreski, Sara Jahnke, Renáta Androvičová, Klára Bártová, Agatha Chronos, Lucie Krejčová, Lenka Martinec Nováková, Kateřina Klapilová

**Affiliations:** 1 Faculty of Humanities, Charles University, Pátkova 2137/5, Prague, 182 00 Czech Republic; 2 Centrum pro sexuální zdraví a intervence, National Institute of Mental Health, Klecany, Czech Republic; 3 Faculty of Psychology, Universitetet i Bergen, Bergen, Norway

**Keywords:** Parental relationship quality, Paraphilia, Rape, Childhood adversity, Agonistic continuum

## Abstract

Research suggests that adverse rearing conditions such as poor-quality relations with one's parents are associated with a range of potentially problematic sexual behaviors and interests. Less is known, however, about the mechanisms linking adverse rearing conditions and paraphilic interest, especially in non-incarcerated individuals. In a representative sample of 1600 Czech adults that oversampled people with paraphilic interests, the current study examined the association between lower quality parental relations and violent paraphilic interest (sexual interest in immobilization, biastophilia, and sadomasochism), and whether this association was mediated by attachment insecurity. Participants completed an online survey with questions assessing violent paraphilic interest, attachment style, and quality of relations with their parents up until age 12. A series of models were fitted to hypotheses based on structural equation modeling. The model estimating a mediation effect of relationship quality with parents on paraphilic interests via attachment insecurity showed an adequate fit to the data (RMSEA = .039, SRMR = .027, CFI = .977, TLI = .953). Controlling for age and gender, lower parental relationship quality was associated with higher levels of violent paraphilic interest and this effect was mediated by insecure attachment, specifically preoccupied and fearful-avoidant attachment styles. Our findings indicate that insecure attachment styles, particularly those that involve anxiety of rejection, may be part of the mechanism linking poor parental bonds and violent paraphilic interest, but the causal nature of these effects cannot be confirmed based on cross-sectional observational data. We discuss study limitations and new research directions in the discussion section.

**Supplementary Information:**

The online version contains supplementary material available at 10.1007/s10508-025-03183-6.

## Introduction

Some people have sexual interests in violent or degrading sexual acts, such as (1) sadomasochism—experiencing and/or inflicting pain or humiliation; (2) biastophilia—rape, particularly of an unknown person; and (3) immobilization—the physical restraint of a non-consenting individual (Bártová et al., 2021; Knight et al., 2013; Longpré et al., 2020; Seto et al., 2025). Rather than representing distinct phenomena, these interests have been hypothesized to be distributed alongside a continuum (sometimes referred to as agonistic continuum based on the Greek term *agonia* meaning struggle or anguish (Knight et al., 2013)). These interests do not inherently lead to harmful sexual behaviors (Joyal & Carpentier, 2022), and some, like sadomasochism, can be practiced with consenting partners (Holvoet et al., 2017). Nevertheless, there is evidence that paraphilic interests in violence may increase the risk of sexual offending. For example, individuals convicted of rape have been found to exhibit a higher degree of physiological sexual arousal by stimuli depicting sexual violence compared to individuals not convicted of sexual offenses (Lalumière & Quinsey, 1994; Lalumière et al., 2006; Seto & Kuban, 1996). Similarly, among the general population, several surveys have found that individuals reporting higher levels of paraphilic interests were more likely to report engaging in corresponding sexual acts, including non-consensual or coercive activities (Bártová et al., 2021; Joyal & Carpentier, 2017; Seto et al., 2021). According to the motivation–facilitation model (Seto, 2019), paraphilic interest involving sexual coercion or non-consensual sadism is important motivational factors for sexual offending against adults. Moreover, paraphilic interest related to sexualized violence is considered a stable factor increasing the chance of sexual recidivism (Mann et al., 2010) and therefore is included in the currently used risk assessment tools (SVR-20; Boer et al., 2017; STABLE-2007, Hanson et al., 2007). While violent paraphilic interests can, in combination with other factors, increase the risk of sexual offending, Brown et al. (2020) conclude that sadomasochistic fantasies and behaviors are not inherently pathological, finding them prevalent (40–70%) and often non-distressing in community samples. It must be emphasized that sadomasochism can be practiced consensually in ways that minimize injury (Dunkley & Brotto, 2020; Holvoet et al., 2017). Individuals interested in consensual sadomasochistic activities are not necessarily drawn to non-consensual ones; indeed, Larue et al. (2014) found only a moderate correlation between consensual and non-consensual sadistic interests. Violent paraphilic interests should thus only be regarded as pathological if they cause significant distress or impairment or lead to serious harm to others.

The etiology of paraphilic interests in violence is little understood. Attachment theory might provide a useful framework to explain the underlying mechanism, as attachment is theorized to be affected by the child’s experiences with their primary caregivers and may play a role in the development of violent sexual fantasies and behaviors (Grady et al., 2017; Maniglio, 2012; Marshall, 1993). This research is still underdeveloped, with most studies focusing on male individuals convicted of sexual offending (ICSO), (e.g., Grady et al., 2021; Lyn & Burton, 2004, 2005; Miner et al., 2010; Smallbone & Dadds, 1998; Ward et al., 1996) which may not generalize to female and non-incarcerated populations. Furthermore, it is still unclear whether attachment style is related to violent sexual behavior or both violent sexual interests and behavior. For example, Grady et al. argue that insecure attachment leads to sexual offending via an increase in"criminogenic needs,"which include"deficits in arousal control, emotion regulation, intimacy and relationship skills, problem solving, self-monitoring, social skills, and victim awareness and empathy, as well as a lack of family support networks, minimal offense responsibility, and sexually unhealthy attitudes"(Grady et al., 2017, p. 438). The potential impact of attachment style on violent paraphilic interest is not explicitly emphasized in Grady et al.’s model. To increase the knowledge base on the etiology of violent paraphilic interests, the present study will assess the link between poor relationship with parents in childhood and violent paraphilic interests in adulthood among a large population-based sample of non-incarcerated men and women from the Czech Republic. Furthermore, it will employ an attachment style framework to investigate the potential mediating role of attachment security and insecurity.

### Adverse Childhood Experiences and Violent Paraphilic Interests

Adverse childhood experiences are characterized by emotional, physical, and/or sexual abuse during childhood as well as poor-quality relations with one's parent. Extensive research suggests that childhood adversity may have long-lasting effects on brain development (Danese & McEwen, 2012; Lupien et al., 2009), health (Felitti et al., 1998), and sexual development (Belsky, 2012). Past research has also established a positive association between exposure to childhood adversity and sexually aggressive behavior (Aebi et al., 2015; Connolly & Woollons, 2008; Dhawan & Marshall, 1996; Jespersen et al., 2009; Knight & Sims-Knight, 2006; Levenson et al., 2016; Pedneault et al., 2020). Accordingly, childhood adversity is an important factor in etiological models of sexual offending (Brouillette-Alarie & Proulx, 2019; Grady et al., 2017; Marshall & Barbaree, 1990; Ward & Beech, 2004) and is included in clinical tools for assessing the risk of sexual offending, such as the SVR-20 (Boer et al., 2017).

There are also a few studies that have examined the association between violent paraphilic interests and childhood adversity in non-incarcerated populations, although findings were inconsistent. In an online survey of the general population, individuals reporting higher levels of childhood physical, emotional, and/or sexual maltreatment also reported greater interest in sadomasochism (Abrams et al., 2022). In contrast, another study did not find a significant difference in childhood physical abuse when comparing BDSM practitioners, non-practitioners with BDSM fantasies, and non-practitioners with no fantasies (Ten Brink et al., 2021). It is important to note that BDSM practitioners may not be representative of all people with violent sexual interests.

### Attachment Style as a Mediator of the Association Between Childhood Adversity and Violent Paraphilic Interests

A putative causal effect of childhood adversity on adult violent paraphilic fantasies may be mediated by a person’s attachment style. Attachment style is a stable, individual pattern of expectations and attitudes about relationships (Bartholomew & Horowitz, 1991; Bowlby, 1982). Traditionally, attachment theorists distinguished between secure attachment and three types of insecure attachment, that is, preoccupied attachment, dismissive attachment, and fearful-avoidant attachment, which vary in their internal working models of self and others (Bartholomew & Horowitz, 1991). According to Bartholomew and Horowitz, securely attached individuals are comfortable forming intimate relationships without becoming overly dependent. They have a positive model of self and a positive model of others, meaning they see themselves as worthy of affection and others as reliable and trustworthy. Individuals with preoccupied attachment have a positive model of others but a negative model of self; they do not avoid intimacy but are anxious about abandonment or rejection, and are overly dependent and controlling in relationships. In contrast, those with a dismissive attachment style have a positive model of self but a negative disposition toward others; they are highly independent, do not fear abandonment, but avoid intimacy. Fearful-avoidant individuals have a negative model of both self and others; they desire intimacy, fear rejection, and abandonment, yet are highly mistrustful of others, thus avoiding closeness. Rather than seeing attachment style as discrete categories, more contemporary versions of attachment theory acknowledge that individuals can express varying levels of different styles (Fraley et al., 2015) and that different attachment styles share common features. For instance, insecure attachment develops in response to poor-quality parenting (e.g., harsh, insensitive) (Bowlby, 1982; Hazan & Shaver, 1987; Schoenmaker et al., 2015). Moreover, both preoccupied and fearful-avoidant types involve anxiety of abandonment (i.e., attachment anxiety), and both dismissive and fearful-avoidant involve a mistrust of others (i.e., attachment avoidance) (Fraley et al., 2015). Insecure attachment styles are associated with loneliness (Bernardon et al., 2011) and difficulties in establishing healthy intimate relationships (Li & Chan, 2012; Mikulincer & Shaver, 2012). Research has thus shifted from conceptualizing attachment styles as discrete categories of attachment toward treating attachment styles as interrelated continuums (Fraley et al., 2015).

Integrating previous theories (e.g., Marshall & Barbaree, 1990; Marshall & Marshall, 2000; Ward & Beech, 2006), Maniglio (2012) hypothesizes a pathway by which adverse rearing conditions, particularly poor parental relations, lead to insecure attachment style, which in turn leads to deviant sexual fantasies, and then potentially sexual offending. Specifically, poor parental relations are theorized to engender an insecure attachment style which is then associated with patterns of relationship dysfunction, loneliness, frustration, perceived rejection, and difficulties establishing healthy consensual intimacy. In response to these social difficulties and frustrations, Maniglio (2012) argues that individuals turn to sexual fantasies involving coercion, objectification, or aggression to achieve a sense of “intimacy, power, or control absent from reality” (p. 89). Mangilio’s proposal is largely theoretical, draws heavily on clinical and intuitive judgements, and requires more empirical testing. There are studies providing initial support for aspects of the model. For instance, studies on forensic populations have found that ICSO exhibit a more insecure attachment style relative to individuals convicted of non-sexual offenses (Lyn & Burton, 2004, 2005; Miner et al., 2010; Smallbone & Dadds, 1998; Ward et al., 1996). Grady and Yoder (2024) review evidence linking insecure attachment style, interpersonal challenges, and sexual offending. In the general population, insecure attachment styles are also associated with various aggressive attitudes and behaviors toward others. For example, insecurely attached individuals exhibit higher levels of hostility (Brodie et al., 2019) and a greater tendency to experience pleasure from the suffering of others (Nickisch et al., 2020). In male university students, insecure attachment has been associated with sexually coercive behavior (Smallbone & Dadds, 2000) as well as hostile attitudes toward women, rape myth acceptance, and an increased likelihood of committing rape (Dang & Gorzalka, 2015). While evidence suggests a relationship between attachment style and sexually violent behavior, little is known about the relationship between attachment style and violent paraphilic interest in non-forensic samples. In one study (Ten Brink et al., 2021), non-practitioners with BDSM fantasies scored significantly higher on preoccupied attachment compared to non-practitioners without fantasies, while individuals recruited from BDSM communities exhibited higher levels of secure attachment (but also preoccupied attachment) than the other groups, suggesting different types of attachment may be more relevant to specific violent paraphilia. More studies are needed to clarify whether there is a link between violent paraphilic interests in more diverse populations.

### Present Study

While numerous studies have examined relationships between childhood adversity and violent sexual behavior, it is less clear how adverse rearing conditions relate to violent sexual interest in non-incarcerated men and women, and whether such associations can be partly explained by insecure attachment style. To test this, we conducted an online survey of adults in the Czech Republic. Our hypotheses were as follows:

1. Individuals reporting lower quality parental relations during childhood express higher levels of violent paraphilic interest.
2. The expected association between lower quality parental relations during childhood and violent paraphilic interest is mediated by insecure attachment.

Additionally, we will explore (3) whether and how specific types of attachment styles mediate the link between quality parental relations during childhood and violent paraphilic interest.

## Method

### Participants and Procedure

Participants were recruited as part of the project"Love and Intimacy in the Czech Republic"which took place January through February 2020. The sampling was performed by the sociodemographic agency, STEM/MARK (https://www.stemmark.cz), which drew participants from the Czech National Panel of 55,000 active panelists and the Dialog panel of 10,000 active panelists (https://www.nationalpanel.eu), in compliance with the ICC/ESOMAR International Code of Marketing and Social Research (https://www.esomar.org). Two groups of participants were recruited from these national panels. The first was a general sample of the Czech population. The second was a pre-selected group of individuals screened for high levels of violent paraphilic interest (from here on referred to pre-screened sample). Specifically, participants were included in the pre-screened sample if they selected the two highest agreement scores (i.e., 4 or 5) on at least one of the four paraphilic scenarios described in the measures section. The two samples did not contain the same participants. By combining the two samples, we created an analytic sample based on the general Czech population, oversampling people with rare paraphilic interests. This step was necessary to increase the precision and power of our statistical analyses.

For both the general sample and pre-screened sample, a nationally representative pool of adult Czech citizens was selected to match quotas for specific sociodemographic characteristics of adults in Czechia (region of residence, population of place of residence, gender, age, and education), based on the latest population census of the Czech Statistical Office (2013). All respondents received email invitations containing a link to an anonymous online survey. Table 1 shows the details about both the general sample and pre-screened for their paraphilic interests from the national panel including information about dropout in both groups. Information about the numbers of excluded individuals for the purpose of this study is also included. The final sample for our analyses thus consisted of 1485 participants from the general sample and 115 from the pre-screened sample, resulting in a total of 1600 participants (818 men, 782 [48.88%] women). Participants were 20 to 91 years old (*M* = 50.5, *SD* = 15.9). All participants provided informed written consent. STEM/MARK awards participants with credits for completing surveys which can then be exchanged for rewards.

**Table 1:** Number of individuals included in the general sample and focus group

	General sample (*N*)	Pre-screened sample (*N*)
Target sample size^a^	1600	200
Selected respondents (number of email invitations sent)	3748	5422
Respondents excluded for not completing the survey	578	753
Respondents excluded for not meeting criteria for high violent paraphilic interest (see 2.2.1)	0	2226
Respondents excluded due to sociodemographic quota saturation and target sample fulfillment	67	1255
Number of complete surveys carried out	1616	127
Respondents excluded because they did not live with both parents up to age 12	131	12
Final sample size	1485	115

^a^Target sample size was determined by statistical power requirements and financial constraints. For the pre-screened sample, target sample size was also based on a recent study of the prevalence of paraphilia in the Czech Republic conducted in 2016 (Bártová et al., 2021)

### Measures

The anonymous online surveys were presented in Czech. The questionnaire on attachment style was translated from English into Czech by a professional translator, and back-translations were produced by research psychologists (LMN, KK). The general sample took 37 min on average to complete their survey, while the pre-screened sample took 46 min on average (note that the pre-screened sample received additional questions that were not included in the questionnaire for the general sample). Beyond the variables included in this research, the two surveys covered the following topics: sexual preferences, fantasies, behaviors, sexual milestones, atypical sexual interests, the number of sexual and romantic relations, behaviors and feelings about intimate relationships and early life experiences. The pre-screened sample answered additional questions about help-seeking, mental health, sexual offense-supportive cognitions, stigma, and hostility toward women. Previous studies based on the same dataset(s) are published elsewhere (Marečková et al., 2022; Zakreski et al., 2024) (see also Martinec Novakova et al., 2025).

#### Paraphilic Interests in Violence

The level of violent paraphilic interest was assessed using self-report items based on a previous national survey on the prevalence of paraphilias in Czech citizens conducted in 2016 (Bártová et al., 2021). Participants were presented with four different paraphilic scenarios taken from the 10 th revision of International Classification of Diseases (ICD-10; World Health Organization, 2004) and asked to rate how sexually arousing they found the fantasy of each scenario on a 5-point Likert-type scale ranging from 1 (definitely not) to 5 (definitely yes): “Immobilization of an unknown, unsuspecting woman/man (e.g., by using violence) and making any resistance impossible. Does the fantasy of this activity elicit your sexual arousal?"(immobilization);"Chasing and raping an unknown, unsuspecting woman/man. Does the fantasy of this activity elicit your sexual arousal?"(biastophilia); “Sexual preference for sadomasochistic activities involving physical or psychological submission or humiliation of the partner. Does the fantasy of this activity elicit your sexual arousal? (sadomasochism—humiliation), and “Sexual preference for sadomasochistic activities that involve inflicting pain by beating or other forms of torture. Does the fantasy of this activity elicit your sexual arousal?” (sadomasochism—pain). Sadism and masochism were combined to be consistent with the ICD-10. The sadomasochism items could thus include both inflicting pain/humiliation or receiving pain/humiliation. A full description of the items, response format, and instructions, including the Czech version, is found in Table S1. The sadomasochism items were phrased in terms of sexual preference due to an error, while in the other items the pure scenario is described. While this is suboptimal, we proceeded with the investigation because sexual preference and sexual arousal are related constructs and the sadomasochism items correlated with the other violent paraphilic items. The four violent paraphilic interest items were used to screen participants in the pre-screened sample. In contrast, participants were included in the general sample regardless of their response to these items. 7.21% of the general sample (107 individuals) and 100% of the pre-screened sample (115 individuals) scored 4/5 or 5/5 on at least one of the four violent paraphilic interest items. In a previous Czech population-based survey using similar items (Bártová et al., 2021), ratings of the violent paraphilic interests (sexual arousal elicited by the paraphilic scenarios) showed high correlations with corresponding dimensions of preferences and fantasies, and medium to high correlations with pornography use, and (desired) sexual behavior, indicating convergent validity. 

#### Parental Relationship Quality

Participants were asked to rate the quality of their relationship with each parent during the first 12 years of life. These items have been used in previous research (Šaffa et al., 2021). Specifically, participants rated the quality of their relationship with their father (defined as the man who raised them) and their mother (defined as the woman who raised them). Participants rated the quality of each relationship on a 7-point Likert-type scale, ranging from 1 (absolutely negative) to 7 (absolutely positive). Participants selected 0 if the parent was missing. We included only participants who were raised by both a father and a mother. This was decided on the grounds that individuals who were not raised by their mother and father may be difficult to compare to individuals who were raised by both their mother and father. For instance, some individuals raised by one parent may have had high quality relations with that parent, but still be affected by the absence of the other parent.

The pre-screened group, but not the general sample, completed a self-report measure of emotional neglect during the first 16 years of life, specifically the 5-item emotional neglect subscale from the Childhood Trauma Questionnaire short-form (Bernstein et al., 2003). See Zakreski et al. (2024) for a more detailed description of this scale. Scores on the emotional neglect subscale were significantly negatively correlated with both the quality of relationship with the mother (Spearman's *r*(113) = −0.46, *p* < 0.001), and the quality of relationship with the father (Spearman's *r*(113) = −0.40, *p* < 0.001).

#### Attachment Style

Attachment style was assessed using the Relationship Questionnaire (Bartholomew & Horowitz, 1991). Participants were presented with descriptions of four different attachment styles (e.g., for dismissive attachment, “I feel good even without close emotional relationships with other people. It is very important for me to be independent and self-sufficient, and I prefer not to rely on others and they do not rely on me.” See Supplementary Materials for an overview of all items and study materials. One scenario described secure attachment, and the other three described different insecure attachment styles (fearful-avoidant, preoccupied, dismissive). For each scenario, participants rated how well that scenario described them on a 7-point Likert scale ranging from 1 (does not describe me at all) to 7 (describes me very well). Options 2 to 6 were not labeled. According to one review article (Ravitz et al., 2010), the Relationship Questionnaire is widely use and"has adequate reliability and very good face and discriminant validity"(p. 428).

## Statistical Analysis

MATLAB 2021b (Mathworks, Inc.) was used to examine the distribution of variables and their correlations. For variables except gender, we performed Anderson–Darling tests to determine if a particular variable was normally distributed. Spearman's correlation coefficient was used since variables were not normally distributed.

To test our hypothesis, we used structural equation modeling (SEM) with confirmatory factor analysis in Mplus8 (version 1.8.8; Muthén & Muthén, 2017). Three SEMs were created. All models will include age and gender (with the binary options of man vs. woman) as control variables, because they are both associated with differences in violent paraphilic interest (Bártová et al., 2021; Holvoet et al., 2017). Furthermore, in addition to showing higher levels of violent paraphilic interest, younger individuals and males are also more likely to express higher levels of insecure attachment (Chopik et al., 2013; Del Giudice, 2019; Konrath et al., 2014). Model 1 examined whether parental relationship quality directly affects violent sexual interests. This model included two latent variables, 1) parental relationship quality, which was indicated by the quality of relationship with the mother, and quality of relationship with the father, and 2) violent paraphilic interest, which was indicated by sexual interest in immobilization, biastophilia, sadomasochism involving pain, and sadomasochism involving humiliation. We regressed violent paraphilic interest onto parental relationship quality. While the parental relationship quality factor only had two indicators, according to Bollen (1989), two-indicator factors are acceptable when the SEM includes more than one factor, and the two-indicator factor correlates with the other factors in the model, as is the case here. Furthermore, while a smaller number of indicators per factor may increase the likelihood of improper solutions or misspecification, all SEMs in the study had a positive definite covariance matrix and there were no negative residual variances. Having a large sample size (*N* > 1000), as we do here, reduces the likelihood of issues associated with two-indicator factors (Marsh et al., 1998).

Model 2 examined whether the association between parental relationship quality and violent paraphilic interest was mediated by attachment insecurity. This model included an additional latent variable, attachment insecurity, which was indicated by the four attachment items (secure, fearful-avoidant, preoccupied, dismissive). The SEM regressed attachment insecurity onto parental relationship quality and regressed violent paraphilic interest onto parental relationship quality and insecurity attachment. To test for mediation, we examined the indirect effect of parental relationship quality on violent paraphilic interest via attachment insecurity.

Model 3 explored whether the association between parental relationship quality and violent paraphilic interest is mediated by the absence or presence of specific types of attachment styles (e.g., low levels of secure attachment, or high levels of preoccupied, fearful, or dismissive). This model regressed each attachment style onto parental relationship quality and then regressed violent paraphilic interest onto each attachment style. For each attachment style, we calculated the specific indirect effect of parental relationship quality on violent paraphilic interest. We then used contrasts to determine whether the effect of one attachment style on violent paraphilic interest was greater than the effect of another attachment style. Contrasts were done for each possible pair of attachment styles.

To determine if the associations between violent paraphilic interest, attachment style, and parental care varied according to the type of violent sexual interest, an additional supplemental model (Model 4) was created. Model 4 was the same as Model 3 except it included two factors for violent paraphilic interest. One factor represented the sadomasochism items, which had a greater potential to imply consent, and the other factor represented the two other violent paraphilic interests (biastophilia, immobilization), which are less likely to be interpreted as consensual.

Sexual interest in biastophilia and immobilization were highly correlated (Spearman's *r* = 0.63, *p* < 0.001). The two sadomasochism items (pain and humiliation) were also highly correlated (Spearman's *r* = 0.75, *p* < 0.001). This might be also due to similarities in item formulation (see also Table S1). It is also possible that the two sadomasochism items correlated more strongly with each other than the other violent paraphilic items because they had a greater likelihood of being interpreted as consensual. To adjust for highly similar items, all three SEMs estimated the covariance between the pain and humiliation items, and between the biastophilia and immobilization items. The third and fourth SEM also estimated covariance between secure attachment and the three insecure attachment styles, again to correct for highly similar items. The fourth SEM did not estimate covariance between the pain and humiliation items, and covariance between the biastophilia and immobilization items because the two pairs of items corresponded to separate factors. Even with the addition of participants from the pre-screened sample, the distribution of scores on the four violent paraphilic interest items was skewed, with values ranging between 1.84 and 2.86, and kurtic, with values ranging from 2.24 to 7.68. Parental relationship items were negatively skewed (quality of relationship with the mother =  − 1.62 and quality of relationship with the father =  − 1.10), and the quality of relationship with the mother was also kurtic (2.36). All six of these items violated the Anderson–Darling test. SEMs were therefore fit using robust maximum likelihood estimation. For each SEM, goodness of fit was assessed by the root mean square error of approximation (RMSEA), the standardized root mean square residual (SRMR), the comparative fit index (CFI), and Tucker–Lewis index (TLI). Good model fit is indicated by RMSEA < 0.06, SRMR < 0.06, and CFI > 0.95, and TLI > 0.95 (Hu & Bentler, 1999). CFI and TLI > 0.90 is marginally acceptable (Bentler, 1990; Bentler & Bonett, 1980).

In terms of sample size requirements for SEM, Bentler and Chou (1987) recommend at least 5 observations per parameter. The first model estimated 23 parameters, thus requiring at least 115 observations. The second model with 39 parameters required at least 195 observations. The third model (53 parameters) required 265 observations. The fourth and supplemental model had 59 parameters thus requiring 295 items. With 1600 participants, we therefore exceeded the recommended minimum sample size for all three SEMs.

## Results

### Descriptive Statistics

Table 2 shows the means and standard deviations of all variables, while their corresponding violin plots are provided in Figure S1-S2. Table 3 shows correlations among study variables. Most participants endorsed low levels of interest across different types of violent paraphilias. Only 222 (13.88%) scored 4/5 or 5/5 on at least one of the four violent paraphilic interest items. To explore potential differences between different types of violent paraphilic interests, supplementary analyses revealed that individuals high on sadomasochism items were younger and included fewer men compared to those high on biastophilia or immobilization, with no differences in parental relationship quality or attachment styles. In terms of attachment style, most of the sample endorsed high levels of secure attachment style and low levels of the three insecure attachment styles. For the latent factors parental relationship quality and violent paraphilic interest, all factor indicators significantly loaded onto their respective latent variables and all standardized factor loadings exceeded the recommended minimum of 0.4 in Models 1, 2, 3, and 4 (Hair et al., 2006).

**Table 2:** Mean and standard deviation of study variables

	Men (*n* = 818)	Women (*n* = 782)	Total (*N* = 1600)
Age	50.36 (15.36)	50.55 (16.39)	50.45 (15.87)
Immobilization	1.72 (1.16)	1.41 (0.92)	1.57 (1.06)
Biastophilia	1.42 (0.94)	1.24 (0.75)	1.33 (0.86)
Sadomasochism (humiliation)	1.55 (1.08)	1.37 (0.91)	1.46 (1.01)
Sadomasochism (pain)	1.40 (0.94)	1.30 (0.84)	1.35 (0.90)
Relationship quality (father)	5.55 (1.68)	5.54 (1.86)	5.54 (1.77)
Relationship quality (mother)	6.15 (1.31)	5.86 (1.60)	6.01 (1.46)
Secure attachment	5.06 (1.95)	5.09 (2.04)	5.07 (1.99)
Fearful-avoidant attachment	3.11 (1.83)	3.24 (2.02)	3.17 (1.93)
Preoccupied attachment	3.09 (1.77)	2.77 (1.83)	2.93 (1.81)
Dismissive attachment	3.15 (2.01)	3.17 (2.10)	3.16 (2.05)

Mean values for each variable. Standard deviations are in parentheses. For the total sample, scores on immobilization, biastophilia, sadomasochism (humiliation), and sadomasochism (pain) scales all ranged between 1 and 5. Scores for parental relationship quality and attachment style scales ranged from 1 and 7

**Table 3:** Correlations between study variables

	1	2	3	4	5	6	7	8	9	10
1. Age										
2. Immobilization	−.22***									
3. Biastophilia	−.13***	.63***								
4. SM pain	−.26***	.51***	.51***							
5. SM humiliation	−.27***	.53***	.49***	.75***						
6. Father Rel. Qual	.15***	−.10***	−.12***	−.14***	−.12***					
7. Mother Rel. Qual	.12***	−.09***	−.13***	−.09***	−.09***	.42***				
8. Secure	.07**	−.10***	−.10***	−.11***	−.12***	.17***	.16***			
9. Fearful-avoidant	−.19***	.17***	.16***	.17***	.15***	−.13***	−.11***	−.38***		
10. Preoccupied	−.16***	.19***	.15***	.17***	.16***	−.14***	−.08***	−.29***	.46***	
11. Dismissive	−.09***	.06*	.06*	.05*	.07**	−.10***	−.08**	−.39***	.21***	.25***

*Rel. Qual.* relationship quality, *SM humiliation* sadomasochism involving humiliation, *SM pain* sadomasochism involving pain, ***p < .001, **p < .01, *p < .05

### Model 1: Parental Relationship Quality and Violent Paraphilic Interest

The model showed borderline good fit (RMSEA = 0.055, SRMR = 0.038, CFI = 0.965, TLI = 0.941) since TLI was just below 0.95. Consistent with past research (Bártová et al., 2021; Holvoet et al., 2017), violent paraphilic interest was higher in younger individuals, β = −0.236, SE = 0.026, *z* = −8.91, *p* < 0.001, and higher in men relative to women, β = 0.122, SE = 0.035, *z* = 3.52, *p* < 0.001. As hypothesized, controlling for age and gender, lower parental relationship quality was associated with significantly higher levels of violent paraphilic interest, β = −0.189, *SE* = 0.049, *z* = −3.86, *p* < 0.001 (see Figure S3 for a depiction of Model 1 with standardized estimates and standard errors).

### Model 2: Does Attachment Security Mediate the Link Between Parental Relationship Quality and Violent Paraphilic Interest

**Fig. 1:**
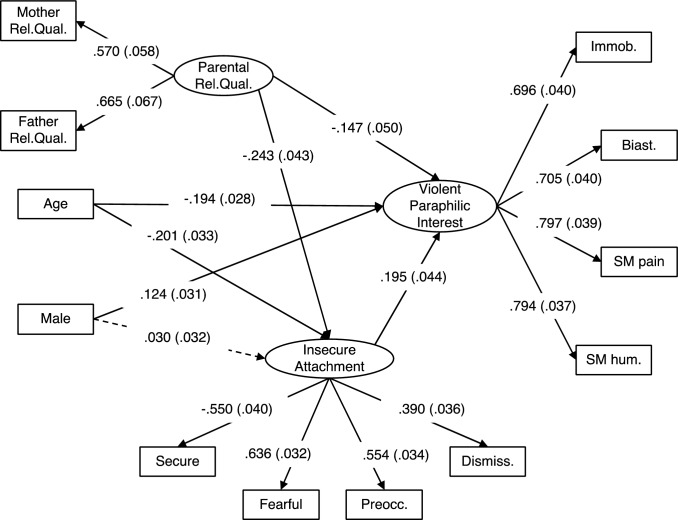
Model 2. SEM examining the effect of parental relationship quality on violent paraphilic interest via attachment insecurity. Standardized estimates are provided with standard error in parentheses. Solid lines are statistically significant associations (*p* <.05). Dashed lines are non-significant. Ovals are latent variables and rectangles are observed variables. Indirect effects and covariance estimates are not shown for the sake of simplicity. Standardized estimates are provided with standard error in parentheses. Biast = biastophilia, Dismiss. = dismissive, Fearful = fearful-avoidant, Immob. = sexual interest in immobilization, Preocc. = preoccupied, Rel.Qual. = relationship quality, SM hum. = sadomasochism involving humiliation, SM pain = sadomasochism involving pain

Figure 1 shows Model 2 with standardized estimates and standard errors. The model showed adequate fit (RMSEA = 0.054, SRMR = 0.042, CFI = 0.937, TLI = 0.911). The insecure attachment latent variable was negatively associated with scores on the secure attachment style item and positively associated with scores on the three insecure attachment style items (fearful-avoidant, preoccupied, and dismissive). Higher levels of this latent variable thus reflect a more insecure attachment style. Standardized factor loadings for secure, preoccupied, and fearful-avoidant attachment exceeded the recommended minimum of 0.4 (Hair et al., 2006), except for dismissive attachment, which was below the recommended minimum (0.39).

Lower parental relationship quality was associated with higher violent paraphilic interest (*z* = −2.91, *p* = 0.004). Lower parental relationship quality was associated with greater attachment insecurity (*z* = −4.37, *p* < 0.001), and greater attachment insecurity was associated with higher levels of violent paraphilic interest (*z* = 5.61, *p* < 0.001). There was also a significant indirect effect of parental relationship quality on violent paraphilic interest via attachment insecurity (β = 0.047, SE = 0.013, *z* = 3.77, *p* < 0.001).

### Model 3: Specific Attachment Styles Mediate Relations between Parental Relationship Quality and Violent Paraphilic Interest

**Fig. 2:**
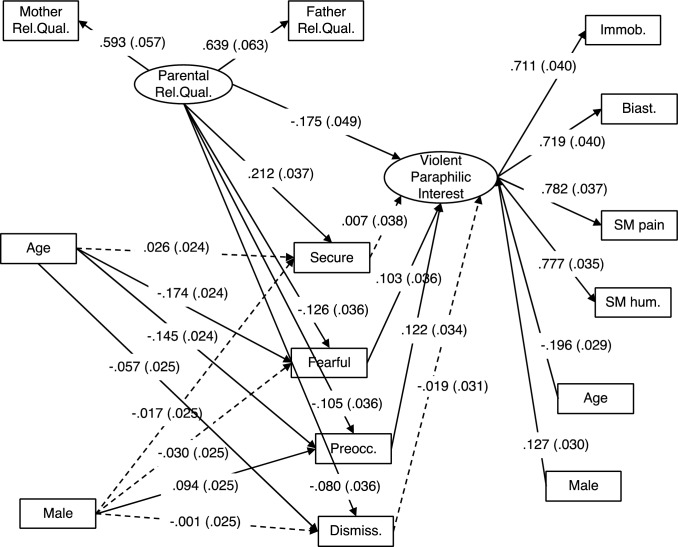
Model 3. SEM examining how the level of specific attachment styles mediate the relationship between parental relationship quality and violent paraphilic interest. Standardized estimates are provided with standard error in parentheses. Solid lines are statistically significant associations (*p* <.05). Dashed lines are non-significant. Ovals are latent variables, and rectangles are observed variables. Standardized estimates are provided with standard error in parentheses. Indirect effects and covariance estimates are not shown for the sake of simplicity. Biast = biastophilia, Dismiss. = dismissive, Fearful = fearful-avoidant, Immob. = sexual interest in immobilization, Preocc. = preoccupied, Rel.Qual. = relationship quality, SM hum. = sadomasochism involving humiliation, SM pain = sadomasochism involving pain

Model 3 demonstrated good fit (RMSEA = 0.039, SRMR = 0.027, CFI = 0.977, TLI = 0.953). In contrast, the previous SEM, which estimated a latent variable based on the four attachment styles, demonstrated only acceptable fit. Figure 2 shows a diagram of this SEM. Lower parental relationship quality was associated with higher levels of violent paraphilic interest (*z* = −3.59, *p* < 0.001). Higher parental relationship quality was associated significantly with higher secure attachment style scores (*z* = 5.78, *p* < 0.001), and lower scores on each of the three insecure attachment style items; fearful-avoidant (*z* = −3.52, *p* < 0.001), preoccupied (*z* =—2.91, *p* = 0.004), and dismissive (*z* = −2.24, *p* = 0.025). Higher levels of violent paraphilic interest were associated with higher levels of fearful-avoidant attachment (*z* = 2.85, *p* = 0.004), and preoccupied attachment (*z* = 3.54, *p* < 0.001). In contrast, violent paraphilic interest was not significantly associated with secure attachment (*z* = 0.18, *p* = 0.852), or dismissive attachment (*z* = −0.59, *p* = 0.552). We then computed contrasts to explore which attachment style had the strongest effect on violent paraphilic interest. The effect of fearful-avoidant attachment was significantly stronger than the effect of secure attachment (*z* = 2.33, *p* = 0.019) and the effect of dismissive attachment (*z* = 2.96, *p* = 0.003), but was not significantly different than the effect of preoccupied attachment (*z* = −0.46, *p* = 0.641). The effect of preoccupied attachment on violent paraphilic interest was significantly stronger than the effect of secure attachment (*z* = 2.61, *p* = 0.009) and the effect of dismissive attachment (*z* = 3.08, *p* = 0.002). The effect of secure attachment on violent paraphilic interest was non-significantly stronger than the effect of dismissive style (*z* = 0.73, *p* = 0.461).

We then examined whether specific attachment styles mediate the effect of parental relationship quality on violent paraphilic interest. The sum of indirect effects (i.e., the total mediating effect) of the four attachment styles was statistically significant (β = −0.023, SE = 0.009, *z* = −2.55, *p* = 0.011). Significant specific indirect effects were observed for fearful-avoidant attachment (β = −0.013, SE = 0.006, *z* = −2.32, *p* = 0.020) and preoccupied attachment (β =—0.013, SE = 0.005, *z* = −2.34, *p* = 0.019). In contrast, the specific indirect effect via secure attachment style was not significant (β = 0.002, SE = 0.008, *z* = 0.18, *p* = 0.852), as was the specific indirect effect via dismissive attachment (β = 0.001, SE = 0.003, *z* = 0.55, *p* = 0.578).

While none of the violent paraphilic items directly specified whether the activities were consensual, the two sadomasochism items (which included terms like"preference"and"partner") may more strongly imply consensual activity compared to the biastophilia and immobilization items. To examine whether the association between parental relationship quality and violent paraphilic interests varied by this potential distinction in perceived consent, we tested a follow-up model (Model 4) separating violent paraphilic interests into two factors: (1) a less consensual interest factor (biastophilia, immobilization) and (2) a sadomasochism factor. Results mirrored Model 3, with both factors showing similar negative associations with parental relationship quality and significant mediation by fearful-avoidant and preoccupied attachment (see Supplemental Materials for full results). This suggests that the broader relationship between parental relationship quality, attachment style, and violent paraphilic interests persists regardless of differences in the potential for consent across the item types.

## Discussion

Using data from an online survey of Czech adults, this study investigated whether the quality of an individual's relationship with their parents during childhood is associated with their level of violent paraphilic interest and whether this association is mediated by attachment insecurity. As hypothesized, individuals reporting poorer quality relations with their parents endorsed significantly higher levels of violent paraphilic interest. This association was partially mediated by a more insecure attachment style, specifically higher levels of fearful-avoidant and preoccupied attachment. These findings contribute to the evidence base in two important ways: (1) Our findings show that adverse parental relations covary with violent paraphilic interests among non-incarcerated men and women (not just in male ICSO), and 2) they provide insight into insecure attachment as a potential mechanism that may link poor-quality parental relations to violent paraphilic interest. Hence, the current study indicates that the link between childhood adversity and violent sexual interests is not an artifact of sampling bias, whereby a correctional sample may include participants with disproportionately high rates of attachment deficits. Furthermore, while violent sexual interests and behaviors may be more prevalent among males (with the exception of masochism which is more common among women), women can also perpetrate sexual violence (Chan, 2023; Russell et al., 2017) and express violent paraphilic interests and behaviors (Bártová et al., 2021; Chan, 2023; Joyal & Carpentier, 2017). While not all individuals with violent sexual interests engage in harmful behaviors, our findings align with several previous studies that have identified an association between violent sexual behavior and adverse childhood experiences such as poor parental relationships, in male ICSO (Connolly & Woollons, 2008; Dhawan & Marshall, 1996; Jespersen et al., 2009; Knight & Sims-Knight, 2006; Levenson et al., 2016) and community samples (Aebi et al., 2015).

Only a few recent studies have examined the relationship between violent sexual interests and childhood adversity in non-incarcerated samples that include both men and women. These studies focused on specific types of violent paraphilic interests (sadomasochism or interest in BDSM), whereas the current study examined interests in sadomasochism, immobilization and biastophilia. All of these interests were moderately or highly positively correlated and loaded on a shared latent factor, lending further support to the concept of the agonistic continuum in a non-English speaking community sample (Longpré et al., 2020). Consistent with our results, Abrams et al. (2022) found a positive association between poor rearing conditions (physical, emotional, sexual maltreatment) and sexual interest in sadomasochism. In contrast, another study (Ten Brink et al., 2021) did not find any differences in childhood physical abuse when comparing BDSM practitioners, non-practitioners with BDSM fantasies, or non-practitioners without BDSM fantasies. Unlike the current study and Abrams et al. (2022), Ten Brink et al. (2021) treated BDSM interest and childhood abuse as categorical variables (yes or no) and did not examine other adverse rearing conditions (such as poor parental relationship quality). A relationship between violent paraphilic interest and childhood adversity may be more apparent when paraphilic interest and childhood adversity are treated as continuous (not categorical) variables. Furthermore, the relationship between childhood adversity and violent paraphilic interest may vary according to the type of childhood adversity. Future research should examine a wider range of adverse childhood experiences and determine how they differentially relate to violent paraphilic interests. It is also possible that associations between violent paraphilic interest, childhood adversity, and attachment might vary between types of violent paraphilic interests (e.g., consensual BDSM vs. non-consensual sadism or biastophilia). In our study, there was no difference between sadomasochistic interests and interests in biastophilia/immobilization in terms of how these two factors relate to parental relationship quality and different attachment styles. We may have observed a difference between these two types of interests if the sadomasochism items explicitly described consensual activity.

Given the pervasive effects of adverse childhood experiences on development (Belsky, 2012; Danese & McEwen, 2012; Lupien et al., 2009), there are multiple potential explanations for the association between poor parental relationship quality and violent paraphilic interest. Our study provides some insight into one potential mechanism that may underlie this association, i.e., attachment style. As hypothesized, poorer quality parental relations were significantly associated with lower levels of secure attachment and higher levels of all three insecure attachment styles (preoccupied, fearful-avoidant, dismissive). While our results cannot establish causality, this finding is consistent with attachment theory (Bowlby, 1982), which argues that warm, responsive, and consistent parenting facilitates the development of secure attachment style, while harsh, cold, or inconsistent parenting leads to an insecure attachment style. While genetics and other environmental factors influence attachment style (Gillath et al., 2008; Sutton, 2019), numerous studies find an association between poor parental relationship quality and emotional neglect during childhood and attachment insecurity in adulthood (Hazan & Shaver, 1987; Müller et al., 2019; Oshri et al., 2015; Schoenmaker et al., 2015).

While parental relationship quality was significantly associated with all four attachment styles, only preoccupied attachment and fearful-avoidant attachment mediated the relationship between low parental relationship quality and higher levels of violent paraphilic interest. Levels of secure attachment style and dismissive attachment style were not significantly associated with violent paraphilic interest. This is in line with the results of Ten Brink et al. (2021) who also found that individuals with BDSM fantasies exhibited higher levels of preoccupied attachment, but did not differ in relation to secure attachment, or dismissive attachment, compared to those without BDSM fantasies. Results from Ten Brink et al. together with our findings suggest that violent paraphilic interest may depend on specific types of insecure attachment. Dismissive attachment style involves a positive view of self and a negative view of others, ultimately leading to avoidance of intimacy (i.e., attachment avoidance) (Bartholomew & Horowitz, 1991). In contrast, preoccupied and fearful-avoidant attachments both involve a negative view of self that is a perception of the self as undesirable or unlovable and consequently the tendency to anticipate and fear rejection (i.e., attachment anxiety) (Bartholomew & Horowitz, 1991). Attachment styles that involve rejection anxiety may thus be more relevant to violent paraphilic interests. One potential reason we speculate is that individuals who view themselves negatively and fear rejection may experience more frustration and be more enticed by the idea of using violence to obtain sexual gratification, as finding a partner who desires them or consents to sex may seem unlikely. At the same time, rejection anxiety may interfere with sexual arousal and pleasure in consensual sexual relations, which could further reinforce a preference for coercive or non-consensual sexual relationships, or sexual relationships where one person dominates or controls the other. Indeed, while both attachment avoidance and attachment anxiety are associated with a range of sexual problems, such as poor sexual performance in men, and in women, poor sexual satisfaction and difficulties achieving orgasm; these difficulties are particularly pronounced among individuals with attachment anxiety (Birnbaum, 2007; Dunkley et al., 2016).

Maniglio (2012) hypothesized that intimacy problems are key in mediating the relationship between insecure attachment styles and violent sexual fantasies and behaviors. This author argues that individuals due to their insecure attachment struggle to establish healthy consensual relationships and consequently experience rejection, loneliness, and frustration. In response to these experiences, individuals may turn to violent sexual fantasies to experience the power and control that are absent from reality. If rejection, loneliness and frustration, and the subsequent desire for power and control are indeed important mechanisms linking insecure attachment and violent sexual interests, this could hypothetically also explain why violent paraphilic interest is more strongly associated with attachment anxiety than avoidance given that individuals with high attachment anxiety are more sensitive to rejection and have a higher need for control, compared to individuals who are high on attachment avoidance (Bartholomew & Horowitz, 1991; Set, 2019) and may experience higher levels of loneliness across relationship domains (Bernardon et al., 2011). In a study of incarcerated men, Hudson and Ward (1997) found that those with preoccupied attachment and fearful-avoidant attachment were lonelier than those with a secure or dismissive attachment style and that fearful-avoidant men reported the greatest hostility toward women.

Our findings suggest violent paraphilic interests are associated with insecure attachment styles involving anxiety (preoccupied and fearful-avoidant attachment) but not dismissive attachment, which is characterized by avoidance of intimacy without anxiety of abandonment (Bartholomew & Horowitz, 1991). In contrast, Ward et al. (1996) found that individuals who sexually offend against children were more likely to exhibit a preoccupied or fearful-avoidant attachment style, while individuals convicted of rape were more likely to show a dismissive attachment style compared to individuals convicted of violent non-sexual offenses. Similarly, a meta-analysis (Karantzas et al., 2016) found that attachment avoidance more so than attachment anxiety was related to the perpetration of sexually coercive behavior. This could mean violent sexual interests and violent sexual behaviors against adults may involve different dimensions of attachment. Violent paraphilic interests may be more closely related to attachment anxiety, whereas attachment avoidance is more closely related to violent or coercive sexual behavior.

Another perspective that may shed light on our findings is Ward et al.’s (1995) theory. Ward et al. argued that preoccupied individuals seek out intimate or sexual relations that they can control, whereas those with a fearful-avoidant attachment are more likely to be sexually aggressive. Indeed, this aligns with our findings of a positive association between violent paraphilic interest and preoccupied and fearful-avoidant attachment. In contrast with our findings, however, Ward et al. argue that individuals with a fearful-avoidant attachment only use sexual coercion in an instrumental way (violence is not part of the pleasure), whereas dismissively attached are sexually aggressive because they are sexually sadistic. In contrast with this, we found no association between dismissive attachment and violent paraphilic interests. Given the inconsistency between our findings and Ward et al.’s theory, future research should revaluate the role of dismissive attachment in violent paraphilic interests and perhaps seek to identify moderating factors.

Since this study is correlational, we cannot conclude that attachment style causes violent paraphilic interests. While early life appears to be a sensitive developmental period for the formation of attachment style, it is also true that later experiences with romantic relationships can alter attachment style (Bayraktaroglu et al., 2023). Individuals with violent paraphilic interests, specifically those that are unable to find receptive partners who share or support such interests, may consequently experience rejection and other poor relationship outcomes which could reinforce an insecure attachment style. Indeed, studies have found that individuals with BDSM interests who are able to find partners or join communities that support such interests in a positive way do not differ from the general population in terms of attachment style (Wismeijer & Van Assen, 2013), sexual problems, or psychological distress (Brown et al., 2020; Richters et al., 2008). In some studies, those individuals actually show better sexual and relationship outcomes (Mundy & Cioe, 2019), and more secure attachment style (Ten Brink et al., 2021) than non-practitioners indicating the presence of moderators or self-selection effects. It therefore important to note that there may be multiple pathways through which poor parental relations, attachment, and violent paraphilic interests are related. For example, rather than being causally related, violent paraphilic interest and attachment style could also covary due to a shared genetic influence. Indeed, research suggests that genes may also influence attachment (Gillath et al., 2008; Sutton, 2019). While we are unaware of studies examining the heritability or genetic correlates of violent paraphilic interest, research does suggest that genetic factors may influence the risk of committing sexual offending (Långström et al., 2015). Future research examining the associations between rearing conditions, attachment, and violent paraphilic interest should take into consideration that biological, psychological, and social variables may play a role in mediating and moderating the connection between these variables.

### Limitations

This study had several limitations which should be addressed in future research. First, participants were limited to men and women. Replication of these findings in gender-diverse populations is needed. Second, we excluded individuals who were not raised by both their mother and father, as it would be difficult to compare their parental relationship quality to individuals who were raised by both their mother and father. This, however, means that our findings may not generalize to individuals who were raised by a single parent or same-sex parents, or different caregivers at different times. Nonetheless, our results should still apply to most current Czech adults. Of our initial participant pool, 8.2% of was excluded as they did not live with both their mother and father. Furthermore, the 2011 EU-SILC survey found that 85.9% of Czech adults (aged 26 to 66) lived with both their mother and father around the age of 14 (as reviewed by Fučík, 2016), though Fučík (2016) notes a rising trend in single parent households and other alternative family structures in the Czech Republic over recent decades. An additional limitation is that, due to ethical and legal obligations acknowledged by the ethical committee, we did not assess whether our participants sexually offended. Due to financial and time constraints, we did not assess whether participants acted on their sexual interests in legal ways. It would be interesting for future research to determine whether the relationship between parental relationship quality, attachment style, and violent paraphilic interest differs between individuals who act on such interests compared to those that do not, or whether there is difference between individuals who engage in consensual BDSM behaviors from those who act on violent sexual interests in non-consensual ways. Future research on the associations between childhood adversity, attachment style, and violent paraphilic interests should try to assess both harmful, illegal behavioral expressions of these interests, as well as consensual BDSM behaviors.

Second, we only conducted a brief assessment of parental relationship quality. Furthermore, since data were collected by self-report measures, social desirability bias may have impacted our results. To reduce socially desirable responding, we collected data via an anonymous online survey to enhance participants’ privacy and encourage accurate responding to sensitive questions (paraphilic sexual interests). Indeed, research indicates that individuals are more inclined to disclose paraphilic interests in online surveys compared to other methods of questionnaire administration (Joyal & Carpentier, 2017). Furthermore, and again due to financial and time constraints, it was not possible to include longer scales with established psychometric properties to assess adults’ recollections of their relationship with their parents or their parents’ behavior, such as the Parental Bonding Inventory (Parker et al., 1979). Recall of past experiences is likely to be affected by memory biases (Baldwin et al., 2019) and memory decay (Jenkins et al., 2002). Additionally, childhood amnesia makes it impossible for adults to recall events from their infant years or early childhood (Tustin & Hayne, 2010). Therefore, retrospective assessments of childhood experiences may lack accuracy due to these cognitive and psychological limitations. This may be particularly relevant when considering the relationship between parental relationship quality and attachment style, as individuals with an insecure attachment style, who have a negative working model of others (Bartholomew & Horowitz, 1991), may have a negative bias when recalling their past relationship with their parents. Recall bias may also affect the observed association between parental relationship quality and violent paraphilic interests. For instance, if an individual was rejected by their family because of their paraphilic interests, they may also recall their early relations with their caregivers as more negative. Ideally, the parent–child relationship should be assessed prospectively (early in life), which would also be important to establish that these relationships pre-date the development of sexual interests in violence. Lastly, we also did not measure specific qualities of parental relationships such as abuse, neglect, or over-controlling or inconsistent parenting. While different violent paraphilic interests were highly correlated in our sample, it is possible that specific aspects of violent paraphilic interest (e.g., dominance, submissiveness, sadism, masochism) are differentially related to attachment style and the quality of parental relationships. Future research should examine how specific aspects of parental relationships contribute to specific aspects of violent paraphilic interest and how this may be mediated by specific attachment styles.

### Conclusion

Poor-quality parental relationships and attachment insecurity are established factors in the etiology of sexual offending. It is less clear how these factors contribute to violent paraphilic interests in non-forensic populations that include both men and women. Drawing from a nationally representative panel of Czech adults, this study found that individuals who self-reported poorer quality relations with their parents exhibited higher levels of violent paraphilic interest and this association was mediated by insecure attachment styles, specifically those involving fear of rejection. Attachment styles that involve a poor working model of the self and anticipation of rejection may therefore be particularly relevant to violent sexual interests in non-incarcerated men and women. Future research is needed to better understand how specific types of insecure attachment relate to violent paraphilic interest and how they affect violent sexual behavior, as well as potential mechanisms that mediate these associations (e.g., consensual relationship problems, and frustration).

## Supplementary Information

Below is the link to the electronic supplementary material.

Supplementary file1 (DOCX 716 KB)

## Data Availability

Data and statistical analysis code have been published to OSF (https://osf.io/ue8j4/?view_only=8002ee5d487043c1b86505938b6f3877, DOI: 10.17605/OSF.IO/UE8J4).
