# Supplementary material for: Preoccupied and Fearful-Avoidant Attachment Styles May Mediate the Relationship Between Poor Parental Relationship Quality and Sexual Interests in Violence
Source: Arch Sex Behav. 2025 Jul 18;54(7):2445–61. doi: 10.1007/s10508-025-03183-6 (PMC12457476; doi:10.1007/s10508-025-03183-6)
Supplement: Supplementary file 1 — Supplementary file1 (DOCX 716 KB) [file 10508_2025_3183_MOESM1_ESM.docx]

**Supplemental Materials**

**Table of Contents**

Demographic and Psychological Differences Between Paraphilia Types ............... 2

Model 4: Separating Sadomasochism from Immobilization and Biastophilia ........ 3

Figure S1 ................................................................................................................. 4

Figure S2 ................................................................................................................. 5

Figure S3 ................................................................................................................. 6

Figure S4 ................................................................................................................. 7

Figure S5 ................................................................................................................. 8

Table S1 ................................................................................................................... 9

**Demographic and Psychological Differences Between Paraphilia Types**

While none of the violent paraphilic items explicitly specified whether the activity was consensual or not, the two sadomasochism items may have a greater potential to imply consensual activity compared to the biastophilia and immobilization items. We therefore compared individuals who scored high (scored 4 or 5) on the sadomasochism items to those who scored high on two less consensual items (biastophilia and immobilization). 60 individuals (28 women, 32 men) scored high on either sadomasochism item without scoring high on the less consensual items. 90 individuals scored high on either biastophilia or immobilization without scoring high on the two sadomasochism items. We then compared the "sadomasochism group" to the "less-consensual group". A chi-square test of independence revealed a significant difference in the proportion of men and women between the two groups (*Χ*² = 4.938, *p* = 0.026), with men comprising 71.11% of the less-consensual items group and 53.33% of the sadomasochism group. According to Welch's *t*-tests, the sadomasochism group was younger (*t* = -2.785, p = 0.007). There were no significant group differences in the quality of relationship with mother (*t* = -0.652, *p* = 0.516), quality of relationship with father (*t* = -1.480, *p* = 0.142), secure attachment levels (t = -1.167, *p* = 0.245), fearful-avoidant attachment (*t* = 0.426, *p* = 0.671), preoccupied attachment (t = 0.914, *p* = 0.362), or dismissive attachment (*t* = 0.745, *p* = 0.458).

**Model 4: Separating Sadomasochism from Immobilization and Biastophilia**

Building on Model 3, we examined whether distinguishing between sadomasochistic interests (humiliation, pain) and less consensual interests (biastophilia, immobilization) would yield different patterns. Figures S4 and S5 show the results of this SEM. Results were consistent with Model 3. The model showed good fit, comparable to Model 3 (RMSEA = .038, SRMR = .025, CFI = .982, TLI = .955). The sadomasochistic factor and less consensual interest factor significantly correlated, *r* = .751, *p* < .001. The two factors were also highly similar in terms of how they related to the other variables in the model. For the less consensual interest factor, higher levels were significantly associated with poorer quality parental relations (*z* = -3.484, *p* < .001), higher levels of fearful-avoidant attachment (*z* = 2.951, *p* = .003), and higher levels of preoccupied attachment (*z* = 3.738, *p* < .001). This factor was not significantly associated with levels of secure attachment (*z* = 0.525, *p* = .600), or dismissive attachment (*z* = -0.605, *p* = .545). The association between poorer quality parental relations and the less consensual interest factor was significantly partly mediated by higher levels of fearful-avoidant attachment (β = -.013, SE = .005, *z* = -2.373, *p* = .018), and higher levels of preoccupied attachment (β = -.013, SE = .006, *z* = -2.370, *p* = .018). In contrast, the specific indirect effect via secure attachment style was not significant (β = .004, SE = .008, *z* = 0.517, *p* = .605), as was the specific indirect effect via dismissive attachment (β = .002, SE = .003, *z* = 0.565, *p* = .572).

The same patterns were observed for the sadomasochism factor. Higher levels of sadomasochistic interest were significantly associated with poorer quality parental relations (*z* = -3.134, *p* = .002), more fearful-avoidant attachment (*z* = 2.413, *p* = .016), and more preoccupied attachment (*z* = 2.830, *p* = .005), but was non-significantly associated with both secure attachment levels (*z* = -0.116, *p* = .908), and dismissive attachment levels (*z* = -0.492, *p* = .623). The relationship between poorer quality parental relations and sadomasochistic interest was partly mediated by fearful-avoidant and preoccupied attachment, but not secure or dismissive attachment levels, with significant indirect effects of fearful-avoidant attachment (β = -.010, SE = .005, *z* = -2.065, *p* = .039), and preoccupied attachment (β = -.010, SE = .005, *z* = -2.075, *p* = .038), and non-significant indirect


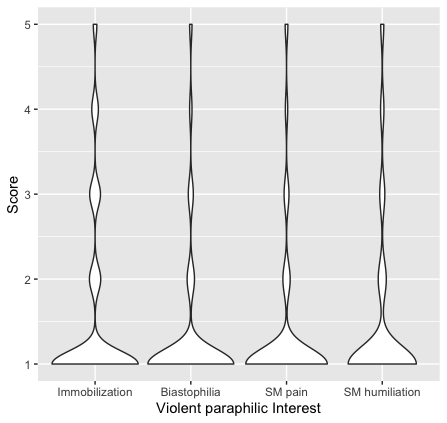


**Figure S1.** Violin plots showing the number of participants selecting each scale option for each type of violent paraphilic interest. For each paraphilic scenario, participants rated how aroused they were by the idea of that scenario on a scale from 1 (definitely not) to 5 (definitely yes). SM = sadomasochism.


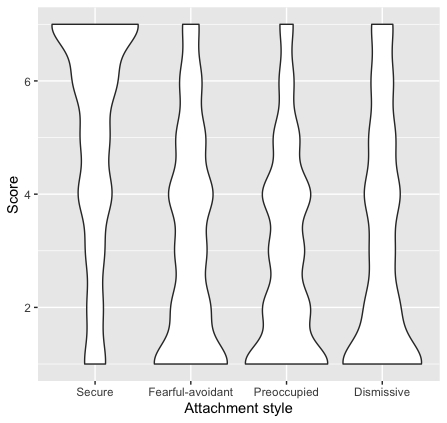


**Figure S2.** Violin plots showing the number of participants selecting each scale option for each attachment style item. For each attachment style, participants rated how well that style described them on a scale from 1 (not at all) to 7 (very much).

**Figure S3**. Model 1. SEM examining the effects of parental care on violent paraphilic interest. Standardized estimates are provided with standard error in parentheses. All lines are statistically significant associations (*p* < .05). Ovals are latent variables and rectangles are observed variables. Standardized estimates are provided with standard error in parentheses. Covariances are not shown for the sake of simplicity. Biast. = biastophilia, Immob. = sexual interest in immobilization, Rel.Qual. = relationship quality, SM hum. = sadomasochism involving humiliation, SM pain = sadomasochism involving pain.

**
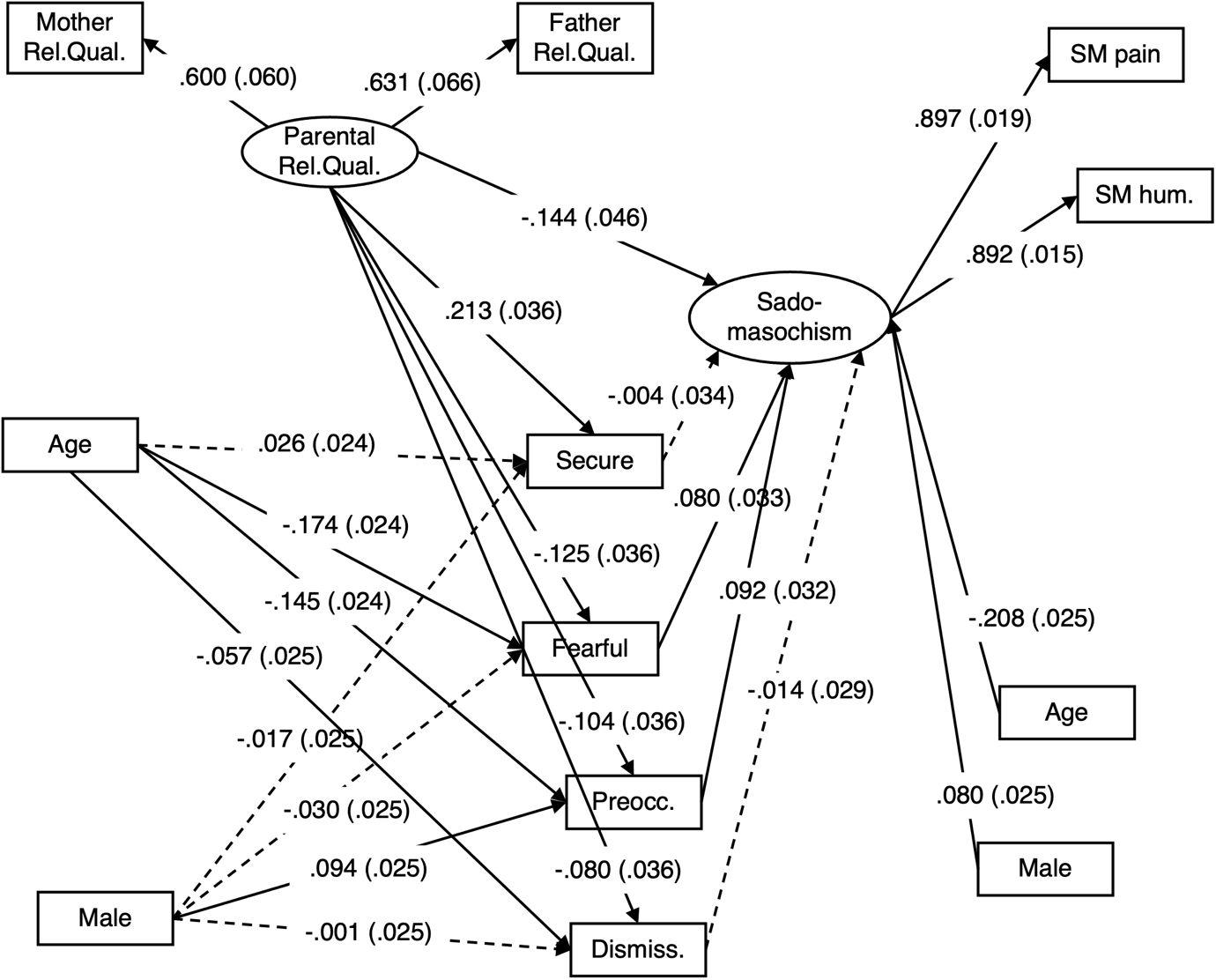
**

**Figure S4.** Model 4 (part showing the sadomasochism latent measure). Violent paraphilic interest was separated into two latent measures (sadomasochism, and less consensual paraphilic interests). To facilitate comprehension, these two factors are shown in two separate figures (Figures S4 and S5). Standardized estimates are provided with standard error in parentheses. All lines are statistically significant associations (*p* < .05). Ovals are latent variables and rectangles are observed variables. Standardized estimates are provided with standard error in parentheses. Covariances are not shown for the sake of simplicity. Biast. = biastophilia, Immob. = sexual interest in immobilization, Rel.Qual. = relationship quality, SM hum. = sadomasochism involving humiliation, SM pain = sadomasochism involving pain.

**
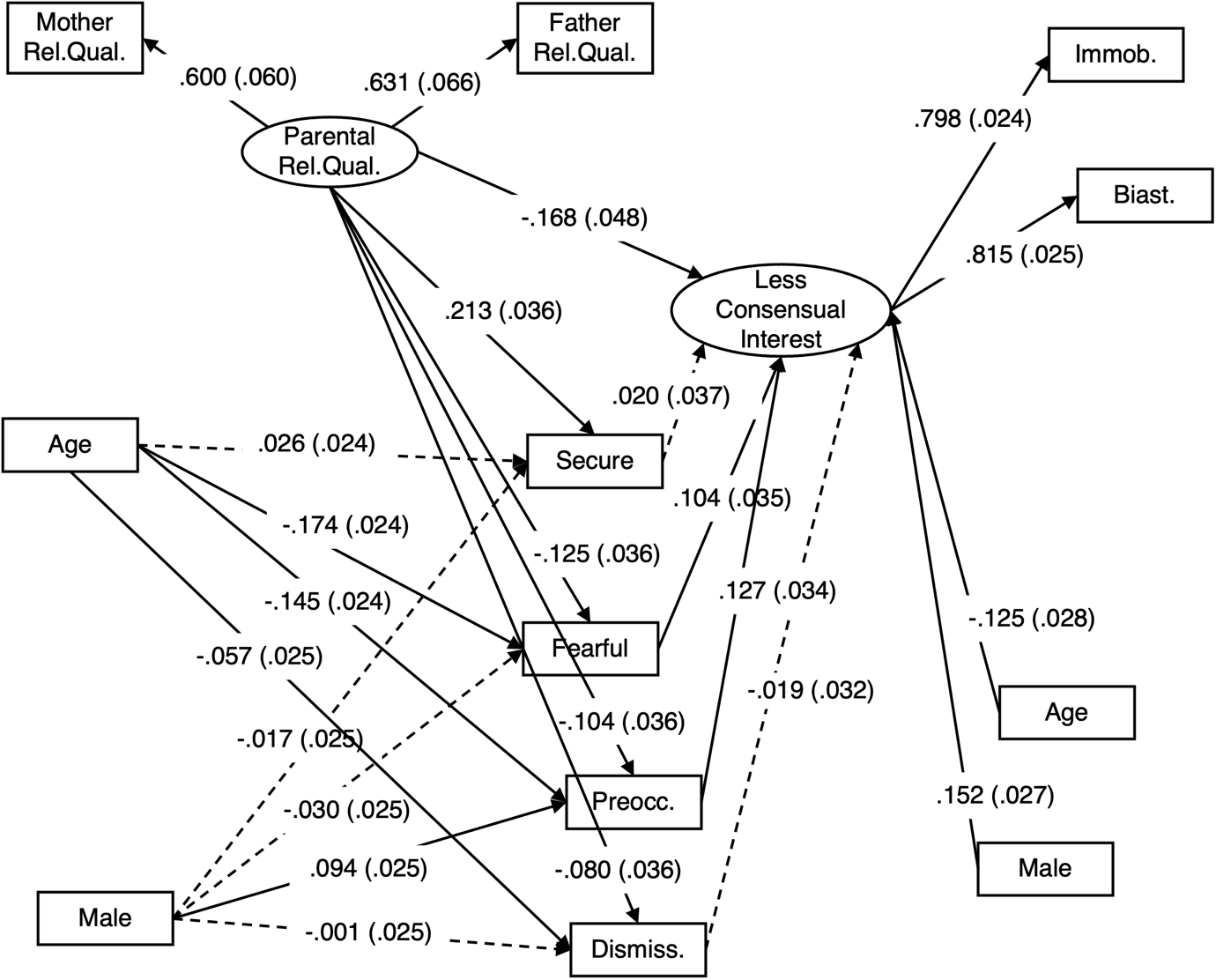
**

**Figure S5.** Model 4 (part showing latent measure representing violent paraphilic interests less consensual). Violent paraphilic interest was separated into two latent measures (sadomasochism, and less consensual paraphilic interests. To facilitate comprehension, these two factors are shown in two separate figures (Figures S4 and S5). Standardized estimates are provided with standard error in parentheses. All lines are statistically significant associations (*p* < .05). Ovals are latent variables and rectangles are observed variables. Standardized estimates are provided with standard error in parentheses. Covariances are not shown for the sake of simplicity. Biast. = biastophilia, Immob. = sexual interest in immobilization, Rel.Qual. = relationship quality.

**Table S1.** *Item instructions, formulations, and response options in Czech with English translations*

| **Scale/subscale** | **Language** | |
| --- | --- | --- |
|  | **English** | **Czech** |
| **Sexual Interest in violence** |  |  |
| Item instruction | In the next part of the survey, you will be presented, besides common sexual activities and partners, with less common sexual patterns that some people may find unpleasant. Other people who have these sexual interests may feel uncomfortable talking about them. Please let us know how you feel about them and provide candid answers using the following scale where a ‘1’ stands for ‘definitely not’ and a ‘5’ means ‘definitely yes’. The survey is anonymous, and the findings will only be used for scientific purposes. Does the idea of this activity sexually arouse you? | V následující části dotazníku se kromě běžně používaných aktivit a sexuálních partnerů objevují i preference neobvyklejší, které mohou být některým lidem nepříjemné. Lidé, kteří je mají, je zase někdy neradi sdělují. Prosíme, odpovězte upřímně, jak to máte Vy a to na následující škále, kdy 1 značí „rozhodně ne“ a 5 značí „rozhodně ano“. Náš dotazník je anonymní a výsledky šetření použijeme pouze pro vědecké účely. Vyvolává ve Vás představa této aktivity sexuální vzrušení? |
| 1) Immobilization | Immobilization of an unknown, unsuspecting woman/man (e.g., by using violence) and making any resistance impossible. Does the fantasy of this activity elicit your sexual arousal? | Znehybnění neznámé, nic netušící ženy/muže (třeba za použití násilí) a znemožnění jejího jakéhokoliv odporu. Vyvolává ve Vás představa této aktivity sexuální vzrušení? |
| 2) Biastophilia | Chasing and raping an unknown, unsuspecting woman/man. Does the fantasy of this activity elicit your sexual arousal? | Pronásledování a znásilnění neznámé, nic netušící ženy/muže. Vyvolává ve Vás představa této aktivity sexuální vzrušení? |
| 3) Sadomasochism-humiliation | Sexual preference for sadomasochistic activities involving physical or psychological submission or humiliation of the partner. Does the fantasy of this activity elicit your sexual arousal? | Sexuální preference pro sadomasochistické aktivity, ve kterých dochází k fyzickému či psychickému podrobení čí ponížení partnera. Vyvolává ve Vás představa této aktivity sexuální vzrušení? |
| 4) Sadomasochism- pain | Sexual preference for sadomasochistic activities that involve inflicting pain by beating or other forms of torture. Does the fantasy of this activity elicit your sexual arousal? | Sexuální preference pro sadomasochistické aktivity, které zahrnují působení bolesti bitím či jinými formami mučení. Vyvolává ve Vás představa této aktivity sexuální vzrušení? |
| Response scale | 1 (definitely not) – 2 – 3 – 4 – 5 (definitely yes) | 1 (rozhodně ne) – 2 – 3 – 4 – 5 (rozhodně ano) |
| **Parental relationship equality** |  |  |
| Instruction | none |  |
| 1) Relationship with father | How do you assess your relationship with your father (the man who raised you) during your childhood (i.e. up to 12 years of age)? | Jak hodnotíte Váš vztah s otcem (mužem, který vás vychovával) během Vašeho dětství (tj. do 12 let)? |
| 2) Relationship with mother | How do you assess your relationship with your mother (the woman who raised you) during your childhood (i.e. up to the age of 12)? | Jak hodnotíte Váš vztah s matkou (ženou, která vás vychovávala) během Vašeho dětství (tj. do 12 let)? |
| Response scale | 1 (absolutely negative) – 2 – 3 – 4 – 5 – 6 – 7 (absolutely positive), 0 (male/female parent did not raise me in childhood or did not raise me until age 12) | 1(Zcela negativně) – 2 – 3 – 4 – 5 – 6 – 7 (Zcela pozitivně), 0 (rodič mužského/ženského pohlaví mne v dětství nevychovával, případně mne vychovával do nižšího věku než 12 let) |
| **Attachment style** |  |  |
| Instruction | In the following section, we will ask you about the type of your relationship with close people. Please read each of the following paragraphs and mark the one that most describes you. Re-read the descriptions of relationships with close people and indicate on the scale to what extent each type describes you.^a^ | V následující části se Vás budeme ptát na typ Vašeho vztahu s blízkými lidmi. Znovu si přečtěte popisy vztahů s blízkými lidmi a na škále zaznačte, do jaké míry Vás každý typ vystihuje. |
| 1) Secure | It is easy for me to experience emotional closeness in my relationship with other people. I feel comfortable when I can rely on others and they can rely on me. I'm not worried that I might be alone or that other people wouldn't accept me. | Je pro mne snadné zažívat ve vztahu s druhými lidmi citovou blízkost. Cítím se příjemně, když se mohu spoléhat na druhé a oni se mohou spoléhat na mne. Nemám obavy, že bych mohl(a) být osamocen(á) nebo že by mne ostatní lidé neakceptovali. |
| 2) Fearful-avoidant | It is difficult for me to experience emotional closeness in my relationship with other people. I would like to have intimate relationships, but I find it hard to trust and rely on other people. I'm afraid I might get hurt if I get too close to other people. | Je pro mne nesnadné zažívat ve vztahu s druhými lidmi citovou blízkost. Chtěl(a) bych mít důvěrné vztahy, ale je pro mne těžké druhým lidem věřit a spoléhat se na ně. Mám strach, že bych mohla být zraněn(a), pokud se příliš přiblížím k ostatním lidem. |
| 3) Preoccupied | I would like to experience true emotional closeness in my relationship with other people, but I feel that others are often reluctant to be as emotionally close to me as I would like. It's uncomfortable for me to be without close relationships, but sometimes I worry that close people won't appreciate me as much as I appreciate them. | Chtěl(a) bych zažívat ve vztahu s druhými lidmi opravdovou citovou blízkost, ale vnímám, že se ostatní často zdráhají být mi tak citově nablízku, jak bych si přál(a). Je pro mne nepříjemné být bez blízkých vztahů, ale někdy se obávám, že by mne blízcí lidé neoceňovali tak, jako oceňuji já je. |
| 4) Dismissive | I feel good even without close emotional relationships with other people. It is very important for me to be independent and self-sufficient, and I prefer not to rely on others and they do not rely on me. | Cítím se dobře i bez blízkých citových vztahů s druhými lidmi. Je pro mne velice důležité být nezávislý/á a soběstačný/á a dávám přednost tomu, abych se nespoléhal(a) na druhé a oni se nespoléhali na mě. |
| Response scale | 1 (it does not describe me at all) – 2 – 3 – 4 – 5 – 6 – 7 (it is very descriptive of me.) | 1 (Vůbec nevystihuje) – 2 – 3 – 4 – 5 – 6 – 7 (Velmi vystihuje) |

*Note*: ^a^ Participants were first presented with descriptions of the four categories and asked to choose which category best described them. They were then presented with the descriptions of the categories again and asked to rate how well each category described them. As explained in the introduction, we choose to conceptualize attachment styles as continuums rather than discrete categories (e.g., Fraley et al., 2015)
